# Horizontal Transmission of Stress Resistance Genes Shape the Ecology of Beta- and Gamma-Proteobacteria

**DOI:** 10.3389/fmicb.2021.696522

**Published:** 2021-07-06

**Authors:** Shady Mansour Kamal, David J. Simpson, Zhiying Wang, Michael Gänzle, Ute Römling

**Affiliations:** ^1^Department of Microbiology, Tumor and Cell Biology, Karolinska Institutet, Solna, Sweden; ^2^Department of Agricultural, Food and Nutritional Science, University of Alberta, Edmonton, AB, Canada

**Keywords:** *Escherichia coli*, *Pseudomonas aeruginosa*, *lebsiella pneumoniae*, Cronobacter *sakazakii*, protease, disaggregase, small heat shock protein, heat tolerance

## Abstract

The transmissible locus of stress tolerance (tLST) is found mainly in beta- and gamma-Proteobacteria and confers tolerance to elevated temperature, pressure, and chlorine. This genomic island, previously referred to as transmissible locus of protein quality control or locus of heat resistance likely originates from an environmental bacterium thriving in extreme habitats, but has been widely transmitted by lateral gene transfer. Although highly conserved, the gene content on the island is subject to evolution and gene products such as small heat shock proteins are present in several functionally distinct sequence variants. A number of these genes are xenologs of core genome genes with the gene products to widen the substrate spectrum and to be highly (complementary) expressed thus their functionality to become dominant over core genome genes. In this review, we will present current knowledge of the function of core tLST genes and discuss current knowledge on selection and counter-selection processes that favor maintenance of the tLST island, with frequent acquisition of gene products involved in cyclic di-GMP signaling, in different habitats from the environment to animals and plants, processed animal and plant products, man-made environments, and subsequently humans.

## Introduction

Bacteria are found in most extreme habitats as these organisms possess an almost unrestricted potential to adapt to altering and adverse environmental conditions including survival of a temporary rise to lethal conditions and occupation of novel ecological niches. Gradual adaptation is mediated by mutations of the core genome, while a rapid and quantum-leap adaptation beyond the functional plasticity of the available genetic repertoire is conferred by mobile genetic elements, plasmids, and transposons, in combination with a vast repertoire of genome engineering tools and repetitive DNA sequences; and the acquisition of novel genes (Shintani, 2017). The horizontally transferred physiological characteristics that are commonly payed attention to include resistance against antimicrobial agents and heavy metals, virulence properties, and widening of catabolic capabilities and resistance (Lan et al., 2001; Elliott et al., 2002; Herold et al., 2004; Lin et al., 2015). Theoretically, and perhaps even practically, there is no restriction to which type of genetic elements are to be horizontally transferred upon exposure to a certain selective pressure; however, properties and transfer of mobile genetic elements that confer resistance to environmental stress (Berendsen et al., 2016) are not as well understood when compared to mobile genetic elements that enhance virulence or mediate antimicrobial resistance.

**FIGURE 1:**
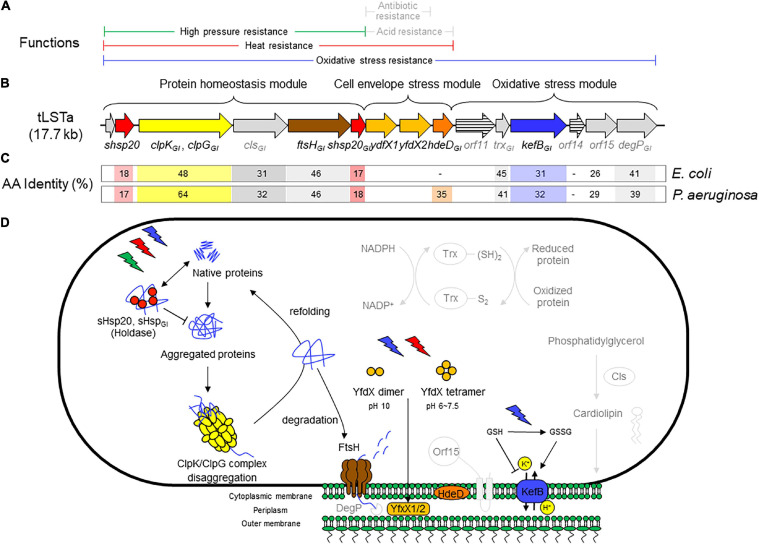
**(A)** Range of chemical or physical stressors that are mitigated by components of the tLST. Stressors are printed in gray if protection is predicted based on the activity of homologous core genome proteins but not for genes encoded by the tLST. **(B)** Schematic representation of the tLSTa and proteins encoded by the genomic island. The length of the open reading frames is drawn to scale. **(C)** Amino acid identity of tLSTa encoded proteins to homologous proteins encoded on the genome of *Escherichia coli* K12 and *Pseudomonas aeruginosa* DSM50071. **(D)** Schematic overview of the role of tLST-encoded proteins in protection against chemical and physical stressors. Pathways are depicted in light gray to indicate phenotypes that are based on *in silico* prediction without experimental confirmation for tLST island encoded proteins. The holding chaperones sHsp20_*GI*_ and sHsp20 prevent irreversible aggregation in an ATP-independent manner and thus work cooperatively with ClpG_*GI*_ (ClpK_*GI*_ in *E. coli*) to prevent or to reverse protein aggregation (Lee et al., 1997, 2015, 2018). FtsH degrades unfolded cytoplasmic, out-of-context and membrane proteins in an ATP-dependent manner and contributes to protein homeostasis in the cytoplasm (Banuett et al., 1986; Tomoyasu et al., 1995; Kamal et al., 2019). YfdX1, YfdX2, and HdeD_*GI*_ act as periplasmic chaperones. YfdX-family proteins were also shown to increase resistance to penicillin G and carbenicillin (Lee et al., 2019), but this activity has not been verified for the tLST encoded YfdX1/2. The chromosomally encoded HdeD improves growth at low pH; this activity was not verified for HdeD_*GI*_ (Liu et al., 2019). Cytoplasmic glutathione (GSH) inhibits KefB activity; oxidation of GSH by chlorine or hydrogen peroxide activates the KefB potassium efflux system which also protects membrane lipids against chlorine-mediated oxidation (MacLean et al., 1998; Wang et al., 2020; Zhu et al., 2021). Cls is a cardiolipin synthase that is responsible for synthesis of membrane lipids (Nishijima et al., 1988). The thioredoxin-dependent reduction system encoded by *trx*_*GI*_ contribute to redox homeostasis (Carmel-Harel and Storz, 2000). The overexpression of *htpX*, the closest homolog to *orf15*, increased the degradation of puromycyl peptides (Kornitzer et al., 1991) and complements FtsH in proteolysis (Sakoh et al., 2005). The core genome DegP is an ATP-independent endopeptidase that degrades periplasmic proteins, functions together with FtsH in proteolysis and is essential for high temperature growth (Lipinska et al., 1990; Nishimura et al., 2016). The genes *orf11* and *orf14* encode proteins that are less than 30% identical to proteins of known function.

A mobile genomic island conferring heat resistance was independently identified in *Escherichia coli* and *Pseudomonas aeruginosa* and termed locus of heat resistance (LHR) and transmissible locus of protein quality control (tLPQC), respectively, (Lee et al., 2015; Mercer et al., 2015). To prevent the continuing use of divergent nomenclature, we propose the term transmissible locus of stress tolerance (tLST). This genomic island provides an exceptional example of the mobilization of a number of highly conserved genes to be commonly horizontally transferred among diverse members of beta- and gamma-Proteobacteria from a so far unknown origin (Lee et al., 2015; Mercer et al., 2015). Initially discovered to mediate tolerance toward lethal heat shock in strains of *Cronobacter sakazakii*, *Klebsiella pneumoniae*, *P. aeruginosa*, and *E. coli* (Bojer et al., 2010; Gajdosova et al., 2011; Lee et al., 2015; Mercer et al., 2015), the tLST island was later identified to provide a wide range of tolerance phenotypes towards environmental and anthropogenic stresses including chlorine and other oxidizing chemicals, and high hydrostatic pressure (Li et al., 2020; Wang et al., 2020) and may interfere with the expression of virulence genes (Wang et al., 2020). The initial analysis of gene products, which are often xenologs of chromosomally encoded genes, showed that tLST island gene products are characterized by physiological and biochemical features that are complementary to, expand or replace the function of core gene products and that allow the organism to enhance persistence and transmission (Wang et al., 2020). The archetype likely close to the major pathogen-related ancestral composite variant of this genomic island is the 18–19 kbp tLSTa (Figure 1), however, several other variants with insertions or deletions have been identified, including the 14–15 kbp tLST1 and the 19 kbp tLST2 (Gajdosova et al., 2011; Lee et al., 2015; Boll et al., 2017; Nguyen et al., 2017). This review aims to summarize current knowledge on the tLST island with respect to ecology, evolution, and mechanisms of resistance. We also use the available information to propose hypotheses related to the evolutionary processes that maintain the tLST island in distinct isolates of many species of Proteobacteria, and the role of the tLST island in the resistance of food-borne and nosocomial bacterial pathogens towards antimicrobial interventions used in food processing, (waste) water treatment, and health care settings.

## Antrophogenic Selective Pressures for the tLST

In 1884, Ferdinand Hueppe described the isolation of the *E. coli* strain C from soured cow’s milk that later became one of the *E. coli* model organisms for biotechnological purposes and basic scientific studies (Hueppe, 1884; Krol et al., 2019). The now available genome sequence revealed that *E. coli* strain C encodes the tLST, suggesting that tolerance against exposure to stress conditions such as oxidative stress and elevated temperatures is a horizontally transferred feature that predates industrial food production, water sanitation, and antibiotic use (Figure 1). Previously documented thermotolerant pathogenic bacteria causing outbreaks due to contaminated milk powder span from an outbreak during the second world war caused by *Salmonella enterica* serovar Senftenberg (Goepfert and Biggie, 1968) to recent infections in neonates caused by *C. sakazakii* (Nazarowec-White and Farber, 1997); both pathogens were later identified to carry the tLST island (Gajdosova et al., 2011; Nguyen et al., 2017; Mercer et al., 2017b). Raw milk contains a diverse bacterial microbiota that is beneficially used for the processing of this animal product to cheese and other fermented products, but may also include pathogenic or opportunistic pathogenic organisms (Quigley et al., 2013). Pasteurization or thermization of fluid and cheese milk may provide an evolutionary pressure to select for organisms that carry the tLST island. This concept is supported by the high prevalence of the tLST island in cheese milk after thermization, i.e., heating to 60°C for 30 min (Marti et al., 2016; Boll et al., 2017). Other anthropogenic habitats with a high prevalence of tLST-positive bacteria include chlorinated waste water, where more than 50% of isolates were found tLST positive (Zhi et al., 2016), North American meat processing plants that employ thermal treatments as pathogen interventions on beef carcasses (Zhang et al., 2020; Guragain et al., 2021), and DaQu, a saccharification starter that is used in China for production of cereal beverages and vinegar (Wang et al., 2018). Daqu is produced from spontaneously fermented cereals; with the fermentation microbiota are recruited from plant microbiota which includes plant-associated Enterobacteriaceae (Zheng et al., 2011). During fermentation, the temperature increases to 50–60°C and fermentation conditions select for Gram-positive bacilli that are heat resistant owing to the presence of the *spoVA^2*mob*^* operon as well as tLST-positive Enterobacteriaceae (Wang et al., 2018).

The presence of the tLST, however, is not limited to bacteria associated with direct anthropogenic manipulation procedures, indicating that efficient horizontal transfer of the tLST to opportunistic pathogenic microorganisms may occur in alternative ecological niche including the environment. The opportunistic pathogen *P. aeruginosa* is foremost an environmental organism that thrives in soil, water and in association with plants (Lee et al., 2020). In *P. aeruginosa*, at least two ubiquitous clones, groups of closely related strains that can be recovered from the clinical habitat including patients as well as the environment have acquired the tLST (Lee et al., 2015). Likewise, the tLST is found in *Cronobacter* and *Klebsiella* species which are relevant as opportunistic and nosocomial (hospital-acquired) human pathogens, but originate from environmental niches (Schmid et al., 2009; Gajdosova et al., 2011; Mercer et al., 2015). Strong selective pressure for maintenance of the tLST may therefore exists also in environmental habitats that can nevertheless be impacted by human activity such as (waste) water treatment.

## The tLST Protects Against Multiple Stresses

The tLST island was originally discovered as it mediated thermotolerance to food-derived bacteria and pathogens (Gajdosova et al., 2011; Lee et al., 2015; Mercer et al., 2015). tLST-mediated thermal tolerance is not incremental but represents a “quantum leap” of superior functionality. For example, the tLST core genes *dna-hsp20-clpG* can provide up to 10-fold higher lethal thermotolerance to genetically unrelated thermosensitive *P. aeruginosa* and *K. pneumoniae* strains (Bojer et al., 2011; Lee et al., 2015). On a similar scale, *E. coli* lacking the entire tLST exhibit a D_60°*C*_-value of less than 1 min while the D_60°*C*_-value of tLST-positive strains of *E. coli* ranges from 10 min to more than 60 min (Li and Gänzle, 2016); i.e., treatment at 60°C for 10 min reduces cell counts of tLST-negative strains by more than 10 log(cfu/mL), while tLST positive strains resist treatment with a reduction of less than 1 log(cfu/mL). tLST-mediated thermotolerance explains the high prevalence of tLST-positive strains of *E. coli* in the meat and cheese production chains (Dlusskaya et al., 2011; Marti et al., 2016; Boll et al., 2017; Zhang et al., 2020; Guragain et al., 2021). tLST-positive strains of *E. coli* also are among the most pressure resistant strains of this species (Liu et al., 2015) and cloning of the tLST island confirmed that the genomic island can increase pressure resistance of *E. coli* (Li et al., 2020). In contrast to heat resistance, pressure resistance in *E. coli* can be conferred by alternative genetic alterations that mediate equivalent pressure resistance in tLST-negative strains (Vanlint et al., 2011).

The observation that tLST-positive strains of *E. coli* are highly enriched in chlorinated waste water (Zhi et al., 2016) led to the discovery that the genomic island also mediates resistance to chlorine, hydrogen peroxide, and peroxyacetic acid but not to acrolein or isothiocyanates (Wang et al., 2020). tLST mediated resistance toward other stressors has not been demonstrated experimentally, but is expected from the predicted protein function. For example, homologs of tLST-encoded proteins were shown to protect against acid stress and to increase antibiotic resistance (Figure 1 and below).

Bioinformatic and functional analyses of the tLST mediated stress resistance suggested that the three different parts of the genomic island predominantly function to protect different segments of the bacterial cells. Proteins encoded by the protein homeostasis module of the tLST (Figure 1) have been shown to predominantly prevent or reverse aggregation and oxidation of cytoplasmic and membrane proteins (Lee et al., 2020). Proteins encoded by the cell envelope stress module are periplasmic chaperones and have been shown to prevent oxidation of membrane lipids. Several proteins of the oxidative stress module are predicted to mitigate oxidative stress by various mechanisms including proteolysis and ion antiport, the latter conducted by KefB_*GI*_. KefB_*GI*_ is a H^+/^K^+^ antiporter that maintains an inside acidic membrane potential at alkaline pH in the presence of chlorine (Figure 1; Mercer et al., 2017a; Lee et al., 2018, 2020; Li et al., 2020; Wang et al., 2020; Zhu et al., 2021). The tLST-mediated pressure resistance phenotype of *E. coli* isolates is provided by the protein homeostasis module (Li et al., 2020); the heat resistance phenotype in *E. coli, K. pneumoniae*, and *P. aeruginosa* is encoded by the protein homeostasis and cell envelope stress modules and *E. coli* strains carrying only the former are substantially less resistant than those carrying both modules or the full island (Bojer et al., 2010; Mercer et al., 2015, 2017a), while the resistance to chlorine and other inorganic oxidative chemicals requires presence of all three modules (Wang et al., 2020). In *P. aeruginosa*, the three genes *dna-shsp20_*GI*_-clpG_*GI*_* on the protein homeostasis module mediate significant thermotolerance to unrelated thermosensitive strains though (Lee et al., 2015).

## Expression of Genes Encoded by the tLST Island

A hallmark of the expression of gene products of the tLST island is their production during exponential growth, which is substantially increased upon entry in the stationary phase of growth at environmental or body temperature (Mercer et al., 2017a) or exclusively highly produced in the late growth phase (Lee et al., 2015). Even more, in *E. coli* and *P. aeruginosa*, tLST-encoded proteins can be among the most abundant proteins (Williams, 1995; Sriramulu et al., 2005; Lee et al., 2015, 2018; Kamal et al., 2019; Li et al., 2020). This expression pattern is distinct from homologous core genome heat shock proteins, which are overexpressed upon exposure to sublethal heat stress. Indeed, a network of proteases has recently been shown to rescue growth arrest of *P. aeruginosa* (Basta et al., 2020). Acquisition of tLST proteases might therefore aid recovery of widely distributed *P. aeruginosa* clones from this environmentally relevant physiological status (Bergkessel, 2020). tLST-mediated heat resistance is enhanced by the presence of 4% NaCl but this effect is mediated by accumulation of compatible solutes rather than over-expression of tLST-encoded proteins as addition of up to 4% NaCl did not increase expression from the tLST promotor that is located 63 bps upstream of the *orf1* (alternatively named dna), a Mer-like transcriptional regulator (Figure 1; Pleitner et al., 2012; Mercer et al., 2017a). Expression of genes encoded by the tLST in *E. coli* at alkaline, but not at neutral pH was reported to be repressed by the Cpx two-component regulatory system which mitigates cell envelope stress during growth at alkaline pH (Zhu et al., 2021). The *in silico* prediction of multiple promoters that respond to diverse environmental stimuli suggests that constitutive expression of tLST island encoded proteins is mediated by multiple factors that have not been fully elucidated (Nguyen, 2019). As a trans mediated cross-reactive physiological trait, the tLST mediated protection against oxidative stress and interferes with the induction of prophages carrying genes coding for the Shiga toxin in the late phage protein region production (Marti et al., 2016; Wang et al., 2020).

## Function of Individual Gene Products Encoded on the tLST Island

A major hallmark of the tLSTa is that the majority of the gene products are xenologs, distantly related homologs of evolutionary highly conserved core genes from a phylogenetically distant bacterial species (Lee et al., 2015, 2016; Mercer et al., 2015). Gene duplication is known as a concept to widen the physiological and metabolic capabilities of organisms including the human pathogen *Mycobacterium tuberculosis* (Tekaia and Gordon, 1999). With respect to their physiological impact, the preservation of gene products such as the proteases FtsH, HtpX, and DegP, small heat shock protein holding chaperones and the redox protein thioredoxin Trx in all organisms including humans emphasizes a central role of these proteins in basic physiological functions such as protein homeostasis and redox balance that are core survival mechanisms of cellular organisms. An overview of the function of proteins that are encoded by the tLSTa is shown in Figure 1. Several gene products including the small heat shock protein sHsp20_1GI_, the disaggregase ClpG_*GI*_/ClpK_*GI*_ and the protease FtsH_*GI*_ (FtsH2) have been genetically and biochemically characterized (Lee et al., 2015, 2018; Kamal et al., 2019). Information on the distinct function of KefB_*GI*_ is derived from studies with *kefB* deletion mutants of tLST-expressing *E. coli* (Zhu et al., 2021). Information on the function of other tLSTa encoded genes is also derived from the expression of plasmid-encoded (fragments of the) tLST in *E. coli* in combination with *in silico* prediction of protein functions that is based on the function of core-genome homologs. As would be expected for xenologous gene products, the amino acid identity of several tLSTa encoded proteins to core genome proteins in *E. coli* or *P. aeruginosa* is low, and even below 30%, i.e., *orf14* shares less than 30% homology to PsiE and *orf15* shares less than 30% homology to the M48 type protease HtpX. A respective functionality and catalytic activity is, though, predicted based on the conservation of the domain and the respective amino acid signatures (Wanner, 1986). For other proteins including the membrane bound protease FtsH, the periplasmic chaperone protease DegP and thioredoxin Trx, the homology to core genome proteins is below 45%, and the catalytic activity can be reliably predicted based on the conservation of signature amino acids. Details on the regulation of the catalytic activity and the respective substrate specificity need to be unraveled by future experimentation.

### Proteins Encoded by the Protein Homeostasis Module

**FIGURE 2:**
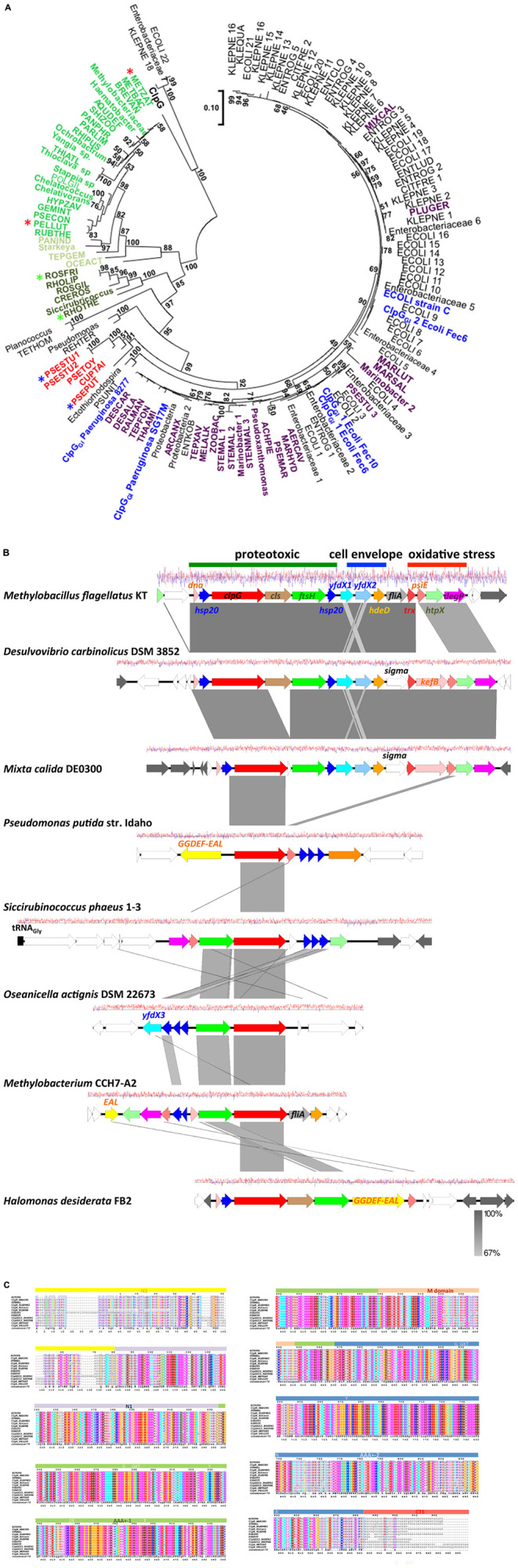
Phylogenetic tree of ClpG_*GI*_ proteins. **(A)** ClpG_*GI*_ proteins most closely related to ClpG_*GI*_ from the urinary isolate *P. aeruginosa* 8277 as query were collected after Blast search with standard parameters at the NCBI hompage (Altschul et al., 1990). Core genome ClpG from *P. aeruginosa* SG17M was used as outgroup. Using the available Blast acquired data, there is a clear distinction in similarity values between ClpG and the ClpG_*GI*_ group family members. However, among the proteins most closely related to ClpG_*GI*_, at least two potentially novel subgroups of ClpG proteins, ClpG2_*GI*_ (in dark green) and ClpH (in green and light green) can be discriminated. ClpG_*GI*_ proteins previously investigated or mentioned in the text in blue, ClpG_*GI*_ proteins from environmental species in violet and an additional potentially distinct novel ClpG_*GI*_ subgroup (ClpG3_*GI*_) in red. Abbreviations used in the tree are defined in Supplementary Table 1. The protein sequences were aligned with ClustalX2.1 (Aiyar, 2000) using standard parameters and in MEGA7 (Kumar et al., 2016), the evolutionary relationship using the standard Maximum Likelihood protocol was determined with 100 bootstraps and the phylogenetic tree subsequently displayed. The branch lengths correspond to the number of substitutions per site. Stars indicate the protein sequences used for the alignment in Figure 2C. **(B)** Gene synteny around representative *clpG*_*GI*_ genes from different *clpG*_*GI*_ subfamilies from archetypical environmental isolates. As a reference, the tLST of the α–proteobacterium *Methylobacillus flagellatus* KT is depicted. The δ-proteobacterium *Desulvovibrio carbinolicus* DSM 3852 encodes a *clpG*_*GI*_ gene member [DESCAR in **(A)**] of the core *clpG*_*GI*_ family closely related to the *P. aeruginosa* clone C *clpG*_*GI*_ gene. On the other hand, *Mixta calida* DE0300 contains a *clpG*_*GI*_ member [MIXCAL in **(A)**] closely related to most *K. pneumoniae* and *E. coli clpG*_*GI*_ genes. *Pseudomonas putida* str. Idaho (PSEPUT), *Siccirubricoccus phaeus* 1–3 (Siccirubricoccus), *Oseanicella actignis* DSM 22673 (OSEACT) and *Methylobacterium* CCH7-A2 (METBAC) encode representatives of the subfamilies ClpG3_*GI*_, a yet undefined subfamily, ClpG2_*GI*_ and ClpH families, respectively. *ClpG*_*GI*_ of *Halomonas desiderata* FB2 is 97.9% identical to *clpG*_*GI*_ of *P. aeruginosa* SG17M. Gene rearrangements and insertion of novel genes occur within tLST. The pathogen-related archetypical composite tLST as depicted in Figure 1 is only present in a subgroup of isolates. While *D. carbinolicus* and γ-proteobacteria *M. calida* possess a close to archetypical tLST, members of the α-proteobacteria such as *O. actignis* DSM 22673, *Methylobacterium* CCH7-A2 and *S. phaeus* 1–3, but also γ-proteobacteria *P. putida* str. Idaho and *H. desiderata* FB2 contain mostly the protein homeostasis part of the tLST gene cluster with an occasional expansion of genes for small heat shock proteins. The gene product of *yfdX3* from *O. actignis* DSM22673 is distinct from *yfdX1* and *yfdX2* and most closely related to a *Paracoccus* representative. As the environmental source, *S. phaeus* 1–3 has been isolated from oil soil, *D. desiderata* from water and *O. actignis* DSM 22673 from the water of a hot spring. The isolation of *Methylobacterium* CCH7-A2 from a hospital shower hose biofilm and *H. desiderata* FB2 and *M. flagellatus* KT from a sewage treatment plant suggests opportunities for horizontal transmission to other microorganisms. *P. putida* str. Idaho is a unique organic solvent tolerant strain. The gene arrangement is centered around the most conserved *clpG*_*GI*_ gene. White colored genes possess core genome or novel functionality, gray colored genes are related to transposition or phage function. The graph reports the G + C content at a window of ±10 nucleotides (red, above average; blue, below average). The figure has been drawn with Easyfig 2.2 (Sullivan et al., 2011). **(C)** As ClpG_*GI*_, the ClpG2_*GI*_, ClpG3_*GI*_, and ClpH proteins are characterized by an AAA +-1 ATPase domain-M-domain-AAA +-2 ATPase domain structure with conserved sequence motifs Walker A and B nucleotide binding motifs, the conserved pore loop residue tyrosine (Y), sensor 1 motifs with conserved threonine (T) and asparagine (N) and sensor 2 motif arginine (R), but possess distinct N (N2 and N1)- and C (CTE) -terminal domains. The alignment was created with ClustalX 2.1 (Aiyar, 2000) and visualized with ESPript 3 (Gross et al., 2007).

The presence of the protein homeostasis module is required for pressure, heat, and chlorine resistance (Mercer et al., 2015; Lee et al., 2016; Li et al., 2020; Wang et al., 2020). The protein homeostasis module includes the two small heat shock proteins sHsp20_1GI_ and sHsp20_2GI_, a cardiolipin synthase, the protease FtsH_*GI*_, and the disaggregase ClpG_*GI*_, which is termed ClpK_*GI*_ in *E. coli* and *K. pneumoniae* (Figure 1). Disaggregases transform aggregated proteins by ATPase driven force into linear and refoldable peptide chains (Lee et al., 2018; Mogk et al., 2018). The ability of the disaggregase ClpG_*GI*_ to process aggregates that are formed at higher temperature or initial protein concentration is superior to the core genome ClpB-DnaK co-disaggregation system (Mogk et al., 1999; Motohashi et al., 1999; Zolkiewski, 1999; Lee et al., 2018). Furthermore, ClpG_*GI*_ directly binds its substrates, protein aggregates, through an extension of the N-terminal domain and displays a high intrinsic ATPase activity (Lee et al., 2018), in contrast to ClpB where the co-chaperone DnaK delivers aggregates to ClpB with subsequent activation of its ATPase activity. *P. aeruginosa* but not *E. coli* has the core genome equivalent ClpG to ClpG_*GI*_ with identical domain structure in addition to the more distantly related disaggregase ClpB. Preliminary one-dimensional protein profiles of respective *P. aeruginosa* SG17M mutants indicated a distinct substrate pattern for each of the three genome encoded disaggregases, ClpB, ClpG, and ClpG_*GI*_ (Lee et al., 2018). Although the phylogenetic origin of ClpG_*GI*_ can be diverse, the ClpG_*GI*_ family consists of highly conserved proteins encoded exclusively by tLST-like gene clusters of pathogens and environmental bacteria of diverse evolutionary origin (Figures 1, 2A,B). The recent information expansion of genome sequences has, however, unraveled several additional ClpG_*GI*_ subgroups that are characterized by distinct N- and C-terminal domains present in various genetic context (Figure 2).

**FIGURE 3:**
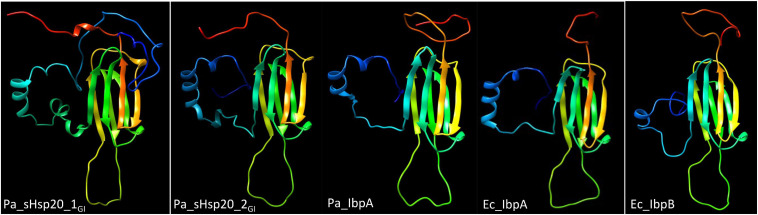
Structural models of sHSP proteins show distinct structures for tLST island and core genome sHsp20 proteins. From left to right, *P. aeruginosa* sHsp20_1GI_, *P. aeruginosa* sHsp20_2GI_, *P. aeruginosa* core genome IbpA, *E. coli* core genome IbpA and *E. coli* core genome IbpB structural model of the monomer. All sHsp20 proteins possess a core of seven antiparallel β-strands that are characteristic for sHsp20 proteins including the paralogous α crystallin reference protein with distinct N-, intramolecular and C-terminal loops. The models were created with Phyre2 (Kelley et al., 2015) based on the structure of the *Triticum aestivum* HSP16.9 protein (1GME chain A; van Montfort et al., 2001) as the best-fit model.

sHsp20 proteins are holding chaperones that prevent irreversible aggregation of proteins in their initial state of unfolding and therefore functionally cooperate with disaggregases (Sun and MacRae, 2005). The core of sHsp20 proteins consists of seven anti-parallel aligned beta-strands. The divergent structurally disordered N- and C-terminal extensions and the intramolecular unstructured loop between the fourth and fifth β-strand of sHsp20 proteins (with the occurrence of up to two sHsp20 proteins on the tLST island) might broaden the substrate range and point to distinct mechanisms of substrate recognition and stabilization equally as subunit homo- and hetero-oligomerization, which is considered the inactive status of sHsp20 proteins (Figure 3; Sun and MacRae, 2005). Of the two sHsp20 proteins encoded by the tLST, only sHsp20_1GI_ has been characterized to provide holding chaperone activity to the thermolabile model substrate citrate synthase (Lee et al., 2015).

The membrane-bound protease FtsH is required for optimal growth and is involved in the turnover of proteins equally as it degrades truncated, out-of-context, and disordered proteins (Banuett et al., 1986; Tomoyasu et al., 1995). Substrate pull-down assays with FtsH proteins that possess a trapping prone ATPase active AAA+ domain, but lack proteolytic activity revealed that the core genome protease FtsH of the aquatic isolate *P. aeruginosa* SG17M distinctively bound and processed substrates compared to FtsH_*GI*_. Selective degradation of PhzC, a key enzyme for the biosynthesis of the redox-active secondary metabolite phenazine and of the heat shock sigma factor RpoH, a major known target of FtsH in *E. coli*, was verified by *in vivo* degradation assays (Kamal et al., 2019). A contribution of the tLSTa-encoded FtsH_*GI*_ to stress tolerance in *E. coli* has not been demonstrated and isogenic strains of *E. coli* carrying tLST2, which encodes a functional version of *ftsH*_*GI*_ and tLST1, were the gene coding for FtsH_*GI*_ is truncated, have comparable heat resistance (Boll et al., 2017). In *P. aeruginosa* clone C, *ftsH*_*GI*_ backs up core genome *ftsH* with regards to a number of phenotypes including heat and antibiotic resistance and biofilm formation (Kamal et al., 2019). The core genomes of *E. coli* and *P. aeruginosa* encode three and four, respectively, cardiolipin synthases with overlapping function (Lee et al., 2020). Cls mediated changes of the composition of membrane fatty acids improved stationary phase survival of *E. coli* (Nishijima et al., 1988; Hiraoka et al., 1993).

### Proteins Encoded by the Cell Envelope Stress Module

The cell envelope stress module encodes the two predicted periplasmic proteins YfdX1 and YfxX2 and the integral membrane protein HdeD_*GI*_. The module is required for heat and chlorine resistance, but not for pressure resistance in *E. coli* (Mercer et al., 2015; Li et al., 2020; Wang et al., 2020). Each of the three proteins of the cell envelope stress module is required for heat resistance (Mercer et al., 2017a; Li et al., 2020). The periplasmic stress chaperones YfdX1 and YfdX2 are predicted to act as periplasmic chaperones to control protein quality. YfdX in *S.* Typhi was also shown to increase resistance to penicillin G and carbenicillin (Lee et al., 2019; Liu et al., 2019) but this activity has not been verified for the tLST encoded YfdX1/2. The chromosomally encoded integral membrane protein HdeD improves growth at low pH in *E. coli* (Mates et al., 2011); however, currently available data do not provide evidence for a contribution of the tLST or the tLST-encoded HdeD_*GI*_ to enhance growth or survival at low pH.

### Proteins Encoded by the Oxidative Stress Module

The function of the oxidative stress module remains poorly characterized when compared to the protein homeostasis and cell envelope stress modules (Figure 1). The oxidative stress module is required for resistance to chlorine but not for heat or pressure resistance. As mentioned earlier, the genes *orf11*, *orf14*, and *orf15* encode proteins that are less than 30% identical to proteins of known function; the expression of the tLHR-encoded *orf15* in *E. coli* too low to be detected by proteome analysis (Li et al., 2020). DegP encoded on the core genome of *E. coli* is a periplasmic chaperone and endopeptidase that is essential for growth at high temperature, aids in protein homeostasis, and activates expression from the alternative sigma factor σ^E^ (Lipinska et al., 1990; Mecsas et al., 1993; Sklar et al., 2007). The *orf16* tLST-encoded DegP is, however, only 41% identical to the core genome DegP; in addition, the tLST island does not improve growth of *E. coli* at high temperature (Ruan et al., 2011) or at alkaline pH (Zhu et al., 2021).

KefbB_*GI*_ is predicted to function as potassium-proton antiporter; this ion exchange acidifies the cytoplasm at alkaline conditions (Elmore et al., 1990). The core genome KefB of *E. coli*, which is 31% identical to KefB_*GI*_, is inhibited by cytoplasmic glutathione and activated by glutathione adducts (Roosild et al., 2010). In accordance with a predicted K^+^/H^+^ antiport activity, the *orf13* gene product KefB_*GI*_ protected *E. coli* against alkaline pH in presence of chlorine, which depletes cytoplasmic glutathione, but not at solely alkaline pH (Zhu et al., 2021).

## Occurrence of tLST or Its Components in Proteobacteria and Other Gram-Negative Bacteria

The tLST with the core genes *dna-hsp20-clpG* encoded in genomes of beta- and gamma-Proteobacteria is generally flanked by mobile elements such as transposases or phage derived genes (Mercer et al., 2015; Lee et al., 2016). Different insertion elements such as IS5, IS3, and Tn*7* elements can flank the tLST (Figure 2B), although the data on complete genomes are too scarce to allow a systematic analysis. The genomic island can be either plasmid or chromosomally encoded; in addition, bacterial genomes can harbor more than one genetically distinct tLST island on the chromosome and on plasmids (Boll et al., 2017; Nguyen et al., 2017; Kamal et al., 2021). The >80% conservation of the tLST on the DNA level throughout even distantly related species suggests a singular and relatively recent source of island mobilization. The G + C content of the tLST is also relatively constant at approximately 61%, whereas the core genomes of its hosts such as *P. aeruginosa*, *E. coli*, *Aeromonas*, *Stenotrophomonas*, and *Acinetobacter* spp. can range from 39 to 70%. This further supports the hypothesis of promiscous horizontal transfer of the genomic island (Supplementary Table 2). However, current data is insufficient to reliably indicate the origin of the genomic island.

**FIGURE 4:**
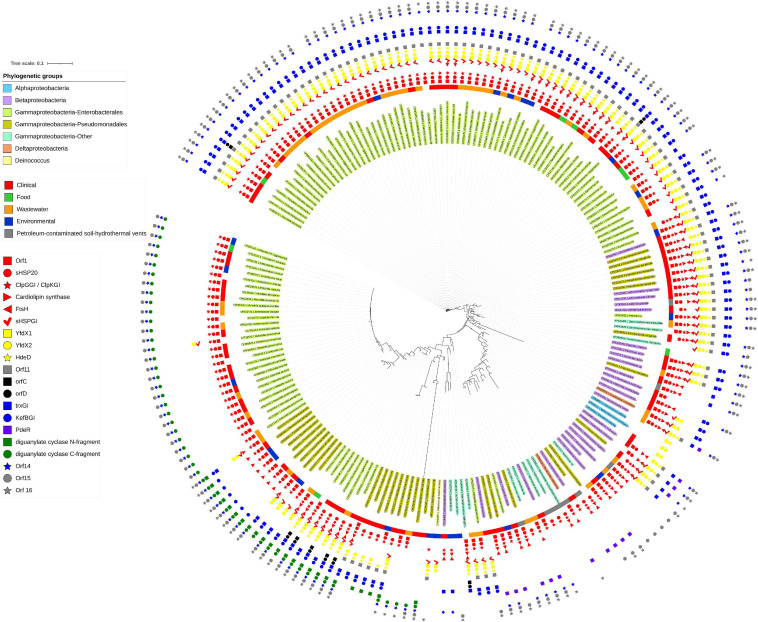
Presence of tLST encoded genes in bacterial genomes. Bacterial genomes containing two or more tLST-encoded genes are color coded as follows (inside to outside): Shading of the bacterial name: taxonomic position; colored ring: source of isolation of the organism; colored symbols indicate the presence or absence of each of the 20 genes encoded by the tLST2 of *S. enterica* serovar Senftenberg. The file was generated with the nucleotide database that was downloaded from NCBI on 20 August 2020. The database was queried using the tLST2 version found in *S. enterica* serovar Senftenberg (CP016838.1:190579-209902) using command line Blastn 2.9.0. All homologous regions which were less than 35 kb and contained more than one tLST gene were extracted (705 sequences) and aligned with Muscle 3.8.1551 (Edgar, 2004). The aligned files were then used to make a tree using FastTree 2.1 (Price et al., 2010). The tree was manually pruned in iTOL (Letunic and Bork, 2019) to remove highly related sequences from the same species and remaining sequences were re-aligned using Mafft 7.407 (Katoh and Standley, 2013). The remaining sequences were also used to create a Blast database, which was queried using the individual tLST genes from *S*. Senftenberg, *Escherichia coli* AW1.7 (GCA_001309455.1) or the PdeR gene from *Pseudomonas* sp. phDV1 (CP031606.1). The presence or absence of the gene was labeled on the tree and truncated tLST versions were inspected to confirm that they were not artifacts of sequencing or assembly. The sources of the strains that contained the tLST sequences used were downloaded from NCBI. The genome accession numbers of the organisms shown and the locations of the tLST sequences in the genomes are indicated in Supplementary Table 3.

The initial description of the archetypical composite tLST in beta- and gamma-Proteobacteria were based on the ∼19 kbp tLSTa (previously: TLPQC) in the water isolate *P. aeruginosa* SG17M, which lacks the cell envelope stress module and adjacent up- and downstream genes (Figure 1; Lee et al., 2015) and the ∼15 kbp tLST1 (previously: LHR1) in food-derived *E. coli*, which lacks the cardiolipin synthase and FtsH_*GI*_ (Mercer et al., 2015). Since 2015, additional tLST variants were described which include insertions in the oxidative stress module and were termed tLST2 (previously: LHR2; Boll et al., 2017; Nguyen et al., 2017). Previous database searches suggested that tLST variants are present mainly in gamma- and beta-Proteobacteria (Mercer et al., 2015). Among the *Enterobacterales*, the tLST has been found virtually exclusive to the *Enterobacteriaceae*. The *Enterobacteriaceae* include environmental or insect-associated bacteria, organisms that are associated with plants but also persist in the intestine of vertebrates and are also of relevance as nosocomial pathogens, e.g., *Klebsiella*, *Enterobacter*, and *Cronobacter* species, and vertebrate- or human-adapted pathogens such as *Shigella* species and *S. enterica* (Adeolu et al., 2016). Remarkably, the tLST has not been detected in insect associated *Enterobacteriaceae* e.g., *Trabulsiella* species (Mercer et al., 2015); and is also absent in the human pathogenic *Shigella* species, *S. enterica*, Shiga-toxin producing *E. coli* and the pandemic ST131 *E. coli* clone. The tLST occurs, however, in non-pathogenic strains of *E. coli* as well as *Klebsiella* and *Cronobacter* species (Mercer et al., 2015; Wang et al., 2020; Kamal et al., 2021). Beyond beta- and gamma Proteobacteria an archetypical composite island is present, for example, in the α-proteobacterium *Methylobacillus flagellatus* KT isolated from a sewage plant (Figure 2B). As a hallmark, a sigma 24 like transcription factor is encoded by the island, but an integrated KefB H^+^/K^+^ transporter is missing (Gajdosova et al., 2011). We further interrogated the distribution of tLST variants by nucleotide BLAST analysis against the NCBI database in August 2020, using each of the genes encoded in the tLST1 or tLST2 as BLAST queries (Figure 4). Genomes that are deposited in the NCBI database predominantly originate from organisms that relate to human activity, particularly human pathogens, and thus do not allow reliable quantification of the distribution of the tLST. To address sampling bias, the NCBI database was used as a qualitative resource and only one sequence variant of the tLST for each bacterial species was randomly chosen (Figure 4). Sequences that include tLST-encoded proteins were predominantly recovered from organisms of the beta- and gamma-Proteobacteria, but alpha- and delta-Proteobacteria and *Deinococcus* of the class *Deinococci* were also represented (Figures 2, 4). Strains encoding for the tLST were isolated not only from anthropogenic sources, i.e., clinical sources, food or food-processing facilities, waste water, and soils contaminated by metal mining or oil extraction, but also from environmental sources including fresh water and hydrothermal vents where the tLST or major components thereof can also reside on a plasmid (Figures 2, 4 and unpublished data citation). Strong selective pressure for transmission and maintenance of the tLST therefore also exists in habitats that are seemingly not impacted by human activity (Figures 2B, 4). Irrespective of sampling bias for genomes deposited in the NCBI database, the high frequency of tLST-positive nosocomial pathogens in conjunction with the virtual absence in human-adapted pathogens such as *Shigella*, *Salmonella* Typhi, and toxin-producing intestinal organisms such as STEC provides evidence to prior suggestions that the tLST does not contribute to the ecological fitness of these pathogens, but can increase virulence or persistence of commensal *E. coli* and organisms in hospitals that are predominantly of environmental origin, but also opportunistic or nosocomial pathogens (Lee et al., 2020; Wang et al., 2020; Kamal et al., 2021).

## Cyclic di-Gmp Second Messenger Signaling Contributes to tLST Island Variability

The tLST1 that encodes for *orf1* (*dna*), *shsp20*_*1GI*_, *clpK*_*GI*_, *shsp20*_*2GI*_, *yfdX1, yfdX2*, *hdeD*_*GI*_, *orf11*, *trx*_*GI*_, *kefB*_*GI*_, *orf14*, *orf15*, and *degP*_*GI*_ has been detected only in *Enterobacteriaceae* (Figure 4 position 11:00 to 2:00). Some of these tLST1 variants include *ftsH*_*GI*_ and individual sequences exclude genes encoded by the oxidative stress module and/or the cell envelope stress module (Figure 4). The tLSTa (Figure 1) is represented mainly by sequences retrieved from beta-Proteobacteria including *Burkholderia* and *Achromobacter* species (Figure 4 at position 2:30). Substantial variability in gene content and amino acid sequence of gene products of the archetypical tLST is found. Several sequences in beta- and gamma-Proteobacteria that are closely related to the tLSTa lack genes of the cell envelope stress module (Figure 4 at position 3:30). The tLST2 (previously: LHR2) which includes the cardiolipin synthase, *ftsH_*GI*_, orfC*, *orfD*, and a diguanylate cyclase has also been exclusively represented by *Enterobacteriaceae* including *E. coli*, *Klebsiella* and *Citrobacter* species and the only strain of *Salmonella* that even harbors two tLST islands, *S. enterica* serovar Senftenberg ATCC 43845 (Figure 4 at position 7:00, Nguyen et al., 2017). Finally, multiple *Enterobacteriaceae* encode a tLST variant termed truncated tLST or tLSTt which encodes only the MerR-like regulator Orf1 (Dna), a putative Dna binding protein; sHSP20_1GI_ and ClpG_*GI*_ of the protein homeostasis module, the C-fragment of the diguanylate cyclase, *orf14*, and *orf15* of the oxidative stress module (Figure 4 at position 8:00 to 10:00). Further more, although tLST gene products are highly conserved, their nevertheless present substantial sequence variability can lead to initial diversification of functional properties and thus provide a study subject for protein evolution in vivo (Kamal et al., 2021).

Genes whose products function as turnover enzymes for the bacterial second messenger cyclic di-GMP can be inserted or replace core tLST genes. For example, tLST variants of *E. coli* carry a disrupted *orf14*, but encode *orfE*, a predicted diguanylase cyclase that has a putative function in starvation survival (Boll et al., 2017), while in another tLST variant from *P. aeruginosa* clone C the gene for the potassium-proton antiporter KefB_*GI*_ is replaced by a putative diguanylate cyclase-phosphodiesterase protein (Lee et al., 2020). Cyclic di-GMP is a ubiquitous second messenger in bacteria that directs the fundamental life style transition between sessility (biofilm formation) and motility as well as between chronic and acute infections (Römling et al., 2013). Recently, the TdcA gene product inserted downstream of the *kefB*_*GI*_ antiporter gene in *P. aeruginosa* was characterized as a thermo-responsive diguanylate cyclase (Almblad et al., 2021). The response to temperature changes is mediated by a Per-Arnt-Sim (PAS) sensory domain, which led to high biofilm formation at body temperature. Increased biofilm formation in response to temperature might thereby aid chronic colonization by *P. aeruginosa*.

In the section above, we emphasized that different modules of the tLST differ in their protection against the various chemical or physical stressors, and protect different segments of bacterial cells (Figure 1). The phylogenetic analysis shown in Figure 4 confirms that this tLST mediated protection against multiple stressors improves the ecological fitness of many organisms, and provides selective pressure for maintenance of those tLST encoded core genes that are present in tLSTa, tLST1, and tLST2 (Figures 1, 4). The high level of expression of tLST encoded proteins (Lee et al., 2015; Kamal et al., 2019; Li et al., 2020) likely also imposes substantial fitness cost, which is apparently offset by the increased resistance to heat, oxidative stress, and additional but yet unknown environmental insults, but can explain the presence of the tLST in only distinct genetic backgrounds of the different species. The gene content of the tLST is altered, however, by deletions or insertions in many bacterial species (Figure 4), indicating that the genomic island is “customized” to match the selective pressures that bacterial species encounter in their respective ecological niches.

## Relevance of the tLST for Nosocomial Pathogens and Antibiotic Resistance

Is the tLST a relevant contributor to persistence of nosocomial pathogens in hospitals and aids their antibiotic resistance? In the section above, we have emphasized that organisms that adapted to an human intestinal pathogenic lifestyle, as is the case for Shiga-toxin producing *E. coli*, *Shigella* species, and *S. enterica*, rarely encode the tLST. Heat resistant and tLST-encoding commensal and meat derived strains of *E. coli* are predominantly of phylotype A, while urinary tract pathogens bearing virulence factors consistently exclude the tLST island consistent with the antivirulence function of the YfdX proteins (Wang et al., 2020; Kamal et al., 2021). The tLST island is frequently present in organisms that are found in environmental or plant-associated niches but can also be nosocomial or opportunistic pathogens (Figure 4; Struve and Krogfelt, 2004; Lee et al., 2017, 2020; Wang et al., 2020). While this pattern is consistent, the mechanisms for tLST maintenance are unknown. However, biofilms of *K. pneumoniae* survive significantly better upon ClpGgi/ClpK production (Bojer et al., 2011) and an endoscope associated outbreak was caused by *P. aeruginosa* clone C strains which usually bear tLST (Fernández-Cuenca et al., 2020). The presence of the tLST in nosocomial pathogens may therefore reduce dispersal limitation, i.e., improve the ability of organisms to survive in water or on surfaces after sanitation, or it may relate to increased virulence upon infection by improved biofilm formation, which can be triggered by thermosensitive cyclic di-GMP modules, or through resistance to antimicrobial compounds generated by immune cells of the host. While a direct contribution of the tLST to antibiotic resistance has not been demonstrated experimentally, the predicted function of several tLST encoded proteins suggests a contribution to the resistance to therapeutic antibiotics (Figure 4).

## Conclusion and Perspectives

The tLST is a composite horizontally transferred genetic element that provides exceptional resistance against a number of clinically and environmentally relevant chemical and physical stressors including heat and oxidative stress. Although those stresses can be occasionally present in a number of ecological niches, selection for tLST positive strains of organisms such as *E. coli*, *P. aeruginosa*, *K. pneumoniae*, and *C. sakazakii* is promoted by human activities including thermal interventions in food processing, hospital-based disinfection procedures and common water and waste water sanitation procedures indicating the need for widening the substrate spectrum for proteases and disaggregases by horizontal transfer of xenologs. The sequence diversity of tLST variants in different bacterial backgrounds implies either adaptation to the host conditions and/or multiple unknown stressors that select for maintenance of the tLST in natural habitats remain unknown, as is the original host for the archetypical tLST from which this composite genomic island was disseminated. Although core activities are well known, the distinct physiological function of multiple tLST encoded proteins remains to be elucidated. In particularly, it is unclear why tLST elements can encode up to three distinct small heat shock proteins (Figure 2B), why multiple variants of the tLST integrate cyclic di-GMP turnover proteins and how these proteins function in stress resistance.

Irrespective of our ability to answer these questions, the tLST appears to contribute to the success of multi-drug-resistant bacteria as nosocomial pathogens and should be considered in efforts to reduce their spread and persistence. Conversely, the demonstrated ability of tLST-encoded proteins to prevent protein aggregation, or to restore aggregated proteins to their native state may be an asset in biotechnological applications that aim to achieve high-yield production of heterologously expressed proteins (Guzzo, 2012). Those characteristics might be extended to clinical applications as ClpG also prevents toxicity of protein substrates involved in neurogenerative diseases (March et al., 2020). Moreover, while core genome heat shock proteins are often essential genes, tLST encoded heat shock proteins are accessory genes and thus provide an excellent tool to study the role of protein homeostasis in bacterial stress resistance and ecology.

## Author Contributions

SK, DS, MG, and UR: conceptualization. SK, MG, and UR: writing. SK, ZW, DS, and UR: data analysis and preparation of figures. SK, DS, ZW, MG, and UR: review and editing of the manuscript. All authors contributed to the article and approved the submitted version.

## Conflict of Interest

The authors declare that the research was conducted in the absence of any commercial or financial relationships that could be construed as a potential conflict of interest.
