# Supplementary material for: Horizontal Transmission of Stress Resistance Genes Shape the Ecology of Beta- and Gamma-Proteobacteria
Source: Front Microbiol. 2021 Jul 6;12:696522. doi: 10.3389/fmicb.2021.696522 (PMC8290217; doi:10.3389/fmicb.2021.696522)
Supplement: Supplementary file 4 [file Table_3.DOCX]

Table S2. List of the accession number, accession ID and the abbreviation on the ClpG_GI_ homologous in the phylogenetic tree.

| **Accession no.** | **Species name** | **Abbreviation on the phylogenetic tree** |
| --- | --- | --- |
| **WP 192284234.1** | *Methylorubrum zatmanii* | METZAT |
| **WP 067253565.1** | *Methylobacterium* sp. CCH7-A2 | METBAC |
| **WP 138141673.1** | *Brevundimonas vancanneytii* | BREVAN |
| **WP 003603277.1** | *Methylobacteriaceae* | Methylobacteriaceae |
| **WP 035714771.1** | *Haematobacter* | Haematobacter |
| **WP 133675726.1** | *Aquamicrobium defluvii* | AQUDEF |
| **WP 160788162.1** | *Shinella zoogloeoides* | SHIZOO |
| **WP 123194513.1** | *Pannonibacter phragmitetus* | PANPHR |
| **WP 155066086.1** | *Paracoccus limosus* | PARLIM |
| **WP 172873192.1** | *Rhizobium pusense* | RHIPUS |
| **WP 038595109.1** | *Ochrobactrum* | Ochrobactrum |
| **WP 164194038.1** | *Yangia* sp. PrR003 | Yangia sp. |
| **WP 038146994.1** | *Thioclava atlantica* | THIATL |
| **WP 078530850.1** | *Thioclava* sp. L04-15 | Thioclava sp |
| **WP 185926889.1** | *Stappia* sp. 28M-7 | Stappia sp |
| **WP 013651411.1** | *Polymorphum gilvum* | POLGIL |
| **WP 026015563.1** | *Chelatococcus* | Chelatococcus |
| **WP 028035861.1** | *Chelativorans* sp. J32 | Chelativorans |
| **WP 020084959.1** | *Hyphomicrobium zavarzinii* | HYPZAV |
| **WP 128490658.1** | *Gemmobacter intermedius* | GEMINT |
| **WP 183754610.1** | *Pseudochelatococcus contaminans* | PSECON |
| **WP 090594962.1** | *Pelagibacterium luteolum* | PELLUT |
| **WP 021096385.1** | *Rubellimicrobium thermophilum* | RUBTHE |
| **CUB01128.1** | *Pannonibacter indicus* | PANIND |
| **CAA0129174.1** | *Starkeya* sp. HF14-78462 | Starkeya |
| **WP 132805264.1** | *Tepidamorphus gemmatus* | TEPGEM |
| **WP 148922259.1** | *Oceanicella actignis* | OCEACT |
| **WP 111471767.1** | *Roseicella frigidaeris* | ROSFRI |
| **WP 201452010.1** | *Rhodovarius lipocyclicus* | RHOLIP |
| **WP 115353464.1** | *Roseomonas gilardii* | ROSGIL |
| **WP 204623660.1** | *Crenalkalicoccus roseus* | CREROS |
| **WP 149539648.1** | *Siccirubricoccus* sp. 1-3 | Siccirubricoccus |
| **WP 128514982.1** | *Rhodobacter thermarum* | RHOTHE |
| **WP 101191947.1** | *Planococcus* | Planococcus |
| **WP 094942879.1** | *Tetzosporium hominis* | TETHOM |
| **WP 074830277.1** | *Pseudomonas* | Pseudomonas |
| **WP 183949261.1** | *Rehaibacterium terrae* | REHTER |
| **ABP80013.1** | *Pseudomonas stutzeri* A1501 | PSESTU_1 |
| **WP 020308826.1** | *Pseudomonas stutzeri* | PSESTU_2 |
| **WP 074914920.1** | *Pseudomonas toyotomiensis* | PSETOY |
| **WP 115713855.1** | *Cupriavidus taiwanensis* | CUPTAI |
| **WP_020190682.1** | *Pseudomonas putida* | PSEPUT |
| **EHQ53701.1** | *Ectothiorhodospira* sp. PHS-1 | Ectothiorhodospira |
| **WP 170860198.1**  **ClpG Pseudomonas aeruginosa** | *Pseudomonas nitroreducens* | PSUNIT |
| **WP 129350687.1** | *Desulfovibrio carbinolicus* | DESCAR |
| **WP 011369123.1** | *Desulfovibrio alaskensis* | DESALA |
| **WP 164491198.1** | *Ralstonia mannitolilytica* | RALMAN |
| **WP 143969071.1** | *Tepidimonas fonticaldi* | TEPFON |
| **WP 004265482.1** | *Thauera aminoaromatica* | THAAMI |
| **WP 009238724.1** | *Proteobacteria* | Proteobacteria |
| **WP 176087783.1** | *Achromobacter anxifer* | ARCANX |
| **WP 016451636.1** | *Proteobacteria* | Proteobacteria_2 |
| **WP 047357390.1** | *Enterobacter kobei* | ENTKOB |
| **WP 133596607.1** | *Tepidicella xavieri* | TEPXAV |
| **WP 019373852.1** | *Melaminivora alkalimesophila* | MELALK |
| **AVZ79854.1** | *Zoogloeaceae bacteirum* Par-f-2 | ZOOBAC |
| **WP 180863296.1** | *Stenotrophomonas maltophilia* | STEMAL_1 |
| **MBH1566135.1** | *Stenotrophomonas maltophilia* | STEMAL_2 |
| **WP 092033903.1** | *Marinobacter* sp. DSM 26671 | Marinobacter |
| **WP 049419568.1** | *Stenotrophomonas maltophilia* | STENMAL_3 |
| **WP 028917703.1** | *Pseudoxanthomonas* sp. J35 | Pseudoxanthomonas |
| **EFF73831.1** | *Achromobacter piechaudii* ATCC 43553 | ACHPIE |
| **WP 126759802.1** | *Pseudidiomarina marina* | PSEMAR |
| **WP 011786805.1** | *Marinobacter hydrocarbonoclasticus* | MARHYD |
| **WP 119197130.1** | *Aeromonas caviae* | AERCAV |
| **WP 021567632.1** | Enterobacteriaceae | Enterobacteriaceae_1 |
| **WP 097471286.1** | *Escherichia coli* | ECOLI_1 |
| **WP 129693901.1** | *Enterobacter roggenkampii* | ENTROG_1 |
| **WP 032174386.1** | Enterobacteriaceae | Enterobacteriaceae_2 |
| **EFJ99472.1** | *Escherichia coli* | ECOLI_2 |
| **ARJ57694.1** | *Escherichia coli* | ECOLI_3 |
| **RFF61962.1** | *Pseudomonas stutzeri* | PSESTU_3 |
| **WP 000101608.1** | Enterobacteriaceae | Enterobacteriaceae_3 |
| **WP 047400210.1** | *Escherichia coli* | ECOLI_4 |
| **MBH92013.1** | *Marinobacter* sp. | Marinobacter_2 |
| **WP 136629548.1** | *Marinobacter salsuginis* | MARSAL |
| **WP 076725024.1** | *Marinobacter lutaoensis* | MARLUT |
| **EGD4990359.1** | *Escherichia coli* | ECOLI_5 |
| **WP 000101612.1** | Enterobacteriaceae | Enterobacteriaceae_4 |
| **WP 115207110.1** | *Escherichia coli* | ECOLI_6 |
| **EFL0496599.1** | *Escherichia coli* | ECOLI_7 |
| **ARH02037.1** | *Escherichia coli* | ECOLI_8 |
| **WP 158127676.1** | *Escherichia coli* | ECOLI_9 |
| **WP 081177954.1** | Enterobacteriaceae | Enterobacteriaceae_5 |
| **EFN4253762.1** | *Escherichia coli* | ECOLI_10 |
| **EFH8032219.1** | *Escherichia coli* | ECOLI_11 |
| **EEW1783696.1** | *Escherichia coli* | ECOLI_12 |
| **EFE7636939.1** | *Escherichia coli* | ECOLI_13 |
| **EMX27879.1** | *Escherichia coli* 174750 | ECOLI_14 |
| **KDX28850.1** | *Escherichia coli* 2-156-04_S4_C2 | ECOLI_15 |
| **WP 000101610.1** | *Escherichia coli* | ECOLI_16 |
| **WP 023329025.1** | Enterobacteriaceae | Enterobacteriaceae_6 |
| **WP 013307886.1** | *Klebsiella pneumoniae* | KLEPNE_1 |
| **WP 064360779.1** | *Pluralibacter gergoviae* | PLUGER |
| **WP 178963130.1** | *Klebsiella pneumoniae* | KLEPNE_2 |
| **GHS68706.1** | *Klebsiella pneumoniae* | KLEPNE_3 |
| **WP 142972739.1** | *Citrobacter freundii* | CITFRE_1 |
| **WP 148731512.1** | *Enterobacter roggenkampii* | ENTROG_2 |
| **WP 193152380.1** | *Enterobacter ludwigii* | ENTLUD |
| **WP 032289797.1** | *Escherichia coli* | ECOLI_17 |
| **WP 192481398.1** | *Escherichia coli* | ECOLI_18 |
| **WP 096037951.1** | *Escherichia coli* | ECOLI_19 |
| **WP 200541043.1** | *Klebsiella pneumoniae* | KLEPNE_4 |
| **WP 048981297.1** | *Klebsiella pneumoniae* | KLEPNE_5 |
| **WP 049129651.1** | *Enterobacter roggenkampii* | ENTROG_3 |
| **WP 145055702.1** | *Mixta calida* | MIXCAL |
| **KTG56833.1** | *Klebsiella pneumoniae* | KLEPNE_6 |
| **SLV57409.1** | *Klebsiella pneumoniae* | KLEPNE_7 |
| **WP 135653465.1** | *Klebsiella pneumoniae* | KLEPNE_8 |
| **WP 080884723.1** | *Klebsiella pneumoniae* | KLEPNU_9 |
| **WP 202072613.1** | *Klebsiella pneumoniae* | KLEPNU_10 |
| **WP 146826945.1** | *Enterobacter roggenkampii* | ENTROG_4 |
| **WP 126347534.1** | *Enterobacter cloacae* | ENTCLO |
| **ARR90204.1** | *Klebsiella pneumoniae* | KLEPNE_11 |
| **SPD96930.1** | *Escherichia coli* | ECOLI_20 |
| **AEV55104.1** | *Klebsiella pneumoniae* | KLEPNE_12 |
| **WP 143114281.1** | *Citrobacter freundii* | CITFRE_2 |
| **BCL43874.1** | *Enterobacter roggenkampii* | ENTROG_5 |
| **GHK32839.1** | *Klebsiella pneumoniae* | KLEPNE_13 |
| **VDB02709.1** | *Klebsiella pneumoniae* | KLEPNE_14 |
| **QKK74692.1** | *Klebsiella pneumoniae* | KLEPNE_15 |
| **WP 096904880.1** | *Klebsiella pneumoniae* | KLEPNE_16 |
| **ONK52358.1** | *Escherichia coli* | ECOLI_21 |
| **WP 102004520.1** | *Klebsiella quasipneumoniae* | KLEQUA |
| **MBC5491427.1** | *Klebsiella pneumoniae* | KLEPNE_17 |
| **EFE6096481.1** | *Escherichia coli* | ECOLI_22 |
| **WP 001619444.1** | Enterobacteriaceae | Enterobacteriaceae_7 |
| **WP 152135180.1** | *Klebsiella pneumoniae* | KLEPNE_18 |
